# Modulation of Autophagy in Cancer Cells by Dietary Polyphenols

**DOI:** 10.3390/antiox10010123

**Published:** 2021-01-16

**Authors:** Claudia Musial, Kamila Siedlecka-Kroplewska, Zbigniew Kmiec, Magdalena Gorska-Ponikowska

**Affiliations:** 1 Department of Medical Chemistry, Medical University of Gdansk, 80-211 Gdansk, Poland; claudia.musial@gumed.edu.pl; 2 Department of Histology, Medical University of Gdansk, 80-211 Gdansk, Poland; kamila.siedlecka-kroplewska@gumed.edu.pl (K.S.-K.); zbigniew.kmiec@gumed.edu.pl (Z.K.)

**Keywords:** autophagy, cancer cells, dietary polyphenols, epigallocatechin gallate, apigenin, oleuropein, punicalgin, resveratrol, pterostilbene, flavonoids

## Abstract

The role of autophagy is to degrade damaged or unnecessary cellular structures. Both in vivo and in vitro studies suggest a dual role of autophagy in cancer—it may promote the development of neoplasms, but it may also play a tumor protective function. The mechanism of autophagy depends on the genetic context, tumor stage and type, tumor microenvironment, or clinical therapy used. Autophagy also plays an important role in cell death as well as in the induction of chemoresistance of cancer cells. The following review describes the extensive autophagic cell death in relation to dietary polyphenols and cancer disease. The review documents increasing use of polyphenolic compounds in cancer prevention, or as agents supporting oncological treatment. Polyphenols are organic chemicals that exhibit antioxidant, anti-inflammatory, anti-angiogenic, and immunomodulating properties, and can also initiate the process of apoptosis. In addition, polyphenols reduce oxidative stress and protect against reactive oxygen species. This review presents in vitro and in vivo studies in animal models with the use of polyphenolic compounds such as epigallocatechin-3-gallate (EGCG), oleuropein, punicalgin, apigenin, resveratrol, pterostilbene, or curcumin and their importance in the modulation of autophagy-induced death of cancer cells.

## 1. Autophagy—Short Introduction

Autophagy, or the mechanism of cell’s self-digestion, is an evolutionarily conserved process used by cells to eliminate senescence or damaged cell organelles as well as damaged or misfolded proteins [1,2,3]. It is upregulated in response to cellular stress such as starvation, oxidative stress, or chemotherapy [1,3]. There are three types of autophagy: microautophagy, macroautophagy, and chaperone-mediated autophagy (CMA) [1,2].

The first of these is the direct uptake of cytosolic components by lysosomal enzymes, which occurs by encircling organelles or parts of cytoplasm by recess of the lysosomal membrane [3,4].

In the mechanism of macroautophagy, autophagic vacuoles are formed such as double membrane-bound autophagosomes, containing cytoplasmic material directed for lysosomal degradation [3,4]. Autophagosomes subsequently fuse with lysosomes to form single membrane-bound autolysosomes, where the sequestrated material is digested by lysosomal enzymes [3,4]. There are a number of steps in the mechanisms of macroautophagy, which influence the formation and function of autophagous vesicles, including their initiation, nucleation, elongation, maturation, and degradation. The described series of processes is closely associated with autophagy-related proteins (ATGs) [3,4].

The initiation of macroautophagy may be related to the presence/conditions of oxidative stress, hypoxia, or the stress resulting from oncological treatment such as chemotherapy and/or radiotherapy [1,2,3].

CMA is characterized by the lysosomal degradation of intracellular proteins. The selective delivery of proteins to lysosomes is mediated by chaperones [2,4,5]. In fact, the presence of vesicles is not required because the substrate during the process reaches the lysosomes via a lysosomal membrane protein translocation complex [2,4,5].

## 2. The Role of Autophagy in Cancer

Autophagy plays a dual role in cancer [6]. Numerous studies conducted over the past 30 years have demonstrated that the impact of autophagy on the development or limitation of the neoplastic process depends on the genetic context, tumor type, tumor stage, microenvironment, and the applied anticancer treatment [7,8,9]. 

Under some conditions, autophagy has a cytoprotective function in cancer. Induction of autophagy in cancer cells resulting in their death can prevent the initiation of tumor growth [6]. Autophagy may also exert anticancer effects by increasing cytotoxicity of the chemotherapeutic agents [10,11,12]. Moreover, effects of autophagy on tumor inhibition or growth depend on the type of tumor [13,14,15]. Some types of cancer may undergo autophagic cell death (ACD) due to the use of some antineoplastic drugs [16]. The mechanism of ACD has been described in *p53*-deficient cancer cells [16]. Available data suggest that the role of autophagy also depends on different stages of tumor development [17]. An example of this is the increased expression of ATG7 as well as down-regulation of *ATG5* and *beclin-1* during apoptosis of colon cancer cells [17]. It was discovered that chloroquine and oxaliplatin interact synergistically on colon cancer cell lines, which confirmed the silencing of ATG5 through RNA interference, whereas the incubation of cells with *beclin-1* resulted in the inhibition of autophagy [17]. 

It has been hypothesized that autophagy may also promote oncogenesis [10]. The primary stage of neoplastic tumor formation is often associated with hypoxia, resulting in metabolic stress [10]. The upregulation of autophagy under these conditions may provide a suitable environment for cancer cells to enter the dormant state. Thus, autophagy may play a pro-survival role by reducing metabolic stress and enabling proliferation of cancer cells [10].

As mentioned, autophagy may also protect tumors from cell death. Some advanced types of tumors are called “autophagous” tumors due to the increased activity of autophagy compared to normal tissues [17]. These tumors include activated Ras tumors and pancreatic cancer [15,16,17]. Moreover, the results clearly indicate that the use of chloroquine, which inhibits autophagy during treatment with bevacizumab, creates better conditions for tumor control and is associated with inhibition of autophagy [17].

Moreover, it has been suggested that autophagy supports survival of dormant tumor cells [1]. This leads to the theory that inhibition of autophagy may be an appropriate therapeutic strategy in the treatment of particular types of tumors [1]. For this aim, autophagy inhibitors which block autophagy at various stages have been used: Unc-51-like Kinase (ULK) inhibitors, Pan PI3Kinhibitors, VPS34 (PI3K) inhibitors, ATG inhibitors (i.e., NSC185058, tioconazol, UAMC-2526, LV320, FMK-9a), lysosomotropic agents, autophagy formation inhibitors (i.e., verteporfin, chloroquine, hydroxychloroquine), inhibitors of vacuolar H+-ATPase (bafilomycin A1), molecules blocking autophagosome-lysosome fusion, acid protease inhibitors, lysosome inhibitors (ionophores, i.e., tambjamines, monensin, and squaramides) and nanoparticles [1,18,19]. 

Cancer stem cells (CSCs) are pluripotent cells that were demonstrated in some types of tumors such as colorectal or breast cancer [20]. They are thought to initiate the development of such tumors being responsible for drug resistance, tumor growth, and recurrence due to their ability to renew themselves and differentiate into various types of tumor cells [21]. It was shown that the process of CMA autophagy (the self-eating process) is a leading factor in both resistance and survival of CSCs [21]. For this reason, it has been suggested that inhibition of autophagy in CSCs may help to overcome their resistance to treatment with anti-cancer drugs [21,22].

Importantly, due to the deregulation of the PI3K/Akt/mTOR signaling pathway in neoplastic cells, autophagy is constitutively activated, which allows for adaptation to the microenvironment and the activation of cell proliferation [23]. The data indicate that cancer therapies should primarily target tumor metabolism due to the induction of autophagy determined by metabolic symbiosis [23]. As a result of the regulation of cellular metabolism, the AMP-induced protein kinase (AMPK) is activated by cancer cells [23,24]. The mechanism of AMPK plays a key role in proliferation, maintains energy homeostasis by regulating cellular metabolism, and inhibits cell growth through phosphorylation by inhibiting the TOR pathway [24]. Moreover, the interaction is also directed towards the stabilization of *p53* and *p27*, i.e., cell cycle inhibitors [24]. It is worth noting that AMPK induces autophagy by stimulating the activation of Ulk1 via phosphorylations of Ser 317 and Ser 777 in a glucose starvation setting [25]. Induction of autophagy as well as U1 regulation signaling has been found to occur through nutrient signaling [25]. 

### 2.1. Autophagy Leading to Cancer Cell Death

Cell death plays a leading role in maintaining proper homeostasis by removing redundant cells [26,27]. In most cases, programmed cell death (PCD) is associated with a caspase-dependent pathway known as apoptosis [26,27]. Importantly, the available data indicate that the different types of programmed cell death pathways can be understood as one integral cell death system [26]. Apoptosis is divided into internal (mitochondrial) pathway, triggered by signals and the developmental program, and external (receptor-dependent) pathway, activated by extracellular ligands [26,27]. Another type of cell death is necrosis, in which the size of the cell increases. Conversely, the most common form of regulated necrosis is necroptosis, for which RIP1 and RIP3 kinase activity is essential. It has been suggested that autophagy may be the specific executive mechanism of necroptosis [28,29].

Autophagy in cell death may be closely related to the simultaneous induction of apoptosis, may occur independently of necrosis and apoptosis, but also may not play an active role in cell death [27,28,29,30].

An autophagy-dependent cell death model has been developed to treat A549 non-small cell lung cancer (NSCLC) lung cancer cells with 200 µM resveratrol (RSV) [31]. The A549 cells were treated with various concentrations of RSV for a period of 48 h [31]. Western blot was used to assess the relative content of an autophagy marker, LC3B protein. Cell death and an increase in autophagic flux were noted, which occurred without apoptosis and necrosis, but required expression of the Atg4B, Atg7, Atg12, and MAP1LC3B genes [31]. In a subsequent experiment [32], A549 cells were incubated with 50, 100, and 200 µM concentrations of RSV. The reduction of viability of A549 cells as a function of RSV dose and time of incubation (24, 48, and 72 h) has been proven. The effect of RSV on A549 autophagy was assessed by RT-qPCR, Western blot analysis, transmission electron microscopy (TEM), and monodansylcadaverine (MDC) staining [32]. The treatment of cells with 50 and 100 µM RSV for 48 h, resulted in the appearance of mature autophagous vacuoles with some of them containing fragments of a damaged endoplasmic reticulum and mitochondria as evidenced by TEM [32]. RT-qPCR analysis showed decreased expression of *p62* mRNA in A549 cells, while expression of *beclin-1* mRNA was increased. 3-methyladenine was used to determine the viability of A549 cells and it was found that resveratrol decreased the viability of the cells [32]. These results clearly indicate that RSV plays a significant role in inducing autophagy in A549 cells [32].

Closely related to autophagic cell death, there is apoptosis and necropotosis, another form of regulated necrotic cell death, ferroptosis, dependent on the intracellular accumulation of iron as well as lipid peroxidation [33]. Moreover, the induction of ferroptosis leads to significant anti-tumor activity. Many studies described the importance of autophagy in the induction of ferroptosis [33]. Importantly, inhibition of ferroptosis can occur through ciclopiroxolamine, 2,2-BP as well as DFA, which are inhibitors of ferroptosis [33]. In addition, proteins such as ferritin are involved in the regulation of the ferroptotic mechanism through iron transport as well as the regulation of metabolism [33,34]. The process of ferroptosis was found to be irreversible after the use of caspase inhibitors [33,34], however, no caspase induction occurs during the ferroptosis process [33,34]. One of the compounds which induce ferroptosis is erastine [34]. Erastine induces *p53* expression or the activity of the RAS-RAF-MEK (RAS protein, rapidly accelerated fibrosarcoma, mitogen-activated protein kinase) pathway, which results in increased activity and production of reactive oxygen species (ROS) [34]. In turn, this mechanism causes, inter alia, the sensitization of cancer cells to the ferroptosis process. Importantly, increasing ER stress activity leads to CCAAT-enhancer-binding protein homologous protein (CHOP)-mediated induction of gene PUMA (*p53* upregulated modulator of apoptosis) expression [34]. As a result of the phosphorylation mechanism of phosphorylated mitogen-activated protein kinase (MAPK), the transcription factor CHOP is activated, which in turn leads to the expression of genes that favors the occurrence of apoptosis. The data show that the occurrence of PUMA activation is mediated by treatment with both a ferroptic agent and TRAIL, which, due to cleavage by caspase 8, leads to apoptosis [34].

### 2.2. Autophagy Promotes Chemoresistance

Chemotherapy resistance is one of the leading causes of cancer recurrence in patients [35]. Autophagy, as mentioned above, may be a trigger for autophagic cell death, but it may also be a protective mechanism of tumor cells that inducing resistance to chemotherapeutic agents [35]. Drug resistance promotes cancer cell survival [35]. The molecular mechanism of autophagy as a response to chemotherapeutic agents is activated via the phosphatidylinositol 3-kinase (PI3K)/AKT/mammalian target of the rapamycin (PI3K/AKT/mTOR) pathway, tumor-suppressor gene *p53*, MAPK14/*p38*α, epidermal growth factor receptor (EGFR), vascular endothelial growth factor (VEGF), and microRNAs (miRNAs) synthesis. Moreover, the mechanisms depend on the type of tumor and treatment methods [35].

There are reports that long non-coding RNAs (lncRNAs) regulate chemoresistance in colorectal cancer (CRC) [36]. One study investigated the role of the RNA 6 lncRNA (SNHG6) gene in CRC chemoresistance in animal models [36] by using flow cytometry, cell drug susceptibility tests, and double luciferase reporter test methods. Autophagic proteins and the SNHG6/miR-26a-5p axis were detected by Western blot and RT-qPCR, which demonstrated the increase in cell resistance to 5-fluorouracil (5-FU) via SNGG6 [36]. Western blot and RT-qPCR indicated the ability of SNHG6 to correlate with ULK1 expression, protein involved in the regulation of autophagy and regulation of miR-26a-5p [36]. The experiment confirmed the enhancement of chemoresistance mediated by SNHG6 in CRC cells [36].

The chemoresistance often occurs during NSCLC treatment [37]. Cis-diamminedichloroplatinum (II) (cisplatin, CDDP) is the most commonly used drug for the treatment for NSCLC [37]. In one experiment, the cisplatin-resistant human lung adenocarcinoma A549 cells (A549/CDDP cell line) and the A549 parental line were used [37]. In order to maintain drug resistance of A549/CDDP cells, 2 mg/mL of cisplatin was added to the medium [37]. Cell viability was determined by the colorimetric method MTS assay. SiRNA transfection, RT, PCR, and Western blot analyses were also performed, while the subcellular localization of antibody LC3B was analyzed by confocal laser scanning microscopy and by TEM [37]. It was found that the induction of autophagy was higher in A549 cells compared to A549/CDDP cells. Moreover, CDDP chemosensitivity was negatively correlated with autophagy activity [37]. According to the investigators, the *beclin-1/ATG5* knockdown increased the induction of cell death following treatment with DDP in the A549/CDDP cell line, indicating a protective role of autophagy due to CDDP treatment via *beclin-1*, knockdown of *ATG5* or inhibition of autophagy via pharmacological platinum-based drugs [37].

## 3. Modulation of Autophagy in Cancer Cells by Polyphenols

Plant-derived polyphenols are divided into two groups, i.e., non-flavonoids and flavonoids (procyanidins) [38]. Both polyphenolic groups exhibit antitumor activity through modulation of non-canonical, i.e., *beclin-1*-independent, and canonical, *beclin*-dependent signaling pathways, as well as regulation of tumor suppressors [38]. The strong antioxidant effect of polyphenols has a significant impact on the modulation of autophagic pathways so that the cell can dispose of defective protein aggregates. Moreover, plant polyphenols were shown to induce the death of neoplastic cells in some model systems [38]. Below, we present a table showing the classification of polyphenols and dietary source (Table 1).

Fruits, vegetables, and herbs are rich in flavonoids. In the current paper, we aim to review the effects of chosen plant-derived polyphenols on autophagy.

### 3.1. EGCG

**Figure 1:**
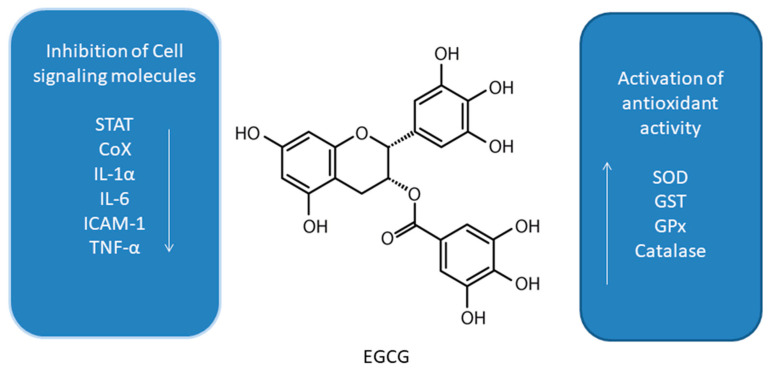
Effect of epigallocatechin-3-gallate (EGCG) on interplay between antioxidant activity and inhibition of cell signaling molecules [42,43,44,45,46,47,48,49,50,51,52,53,54,55,56,57].

Green tea (*Camellia sinesis*) polyphenols, and more specifically main catechin epigallocatechin-3-gallate (EGCG) (Figure 1), have been subjected to a number of studies suggesting induction of autophagy under cell stress conditions in some cancer cell lines [42,44,45]. EGCG was shown to induce autophagosome induction in human hepatoma (HepG2) cells as well as in bovine endothelial cells [44,45]. The cytoprotective mechanism of EGCG was also confirmed in the endoplasmic reticulum (ER) stress response in human embryonic kidney cell line (HEK293T, ATCC, and CRL-3216) [46,47,48]. The experiment revealed a disturbance of the balance of mTOR-AMPK pathways via GADD34 due to the ER stress [48]. Treatment of cells with EGCG, reduces the negative effect of GADD34 inhibition on the autophagy process [48]. The reduction of the negative impact of GADD34 inhibition in relation to autophagy was achieved by treatment with siGADD34 or with guanabenz [48]. In addition, the thapsigargin (TG) interferes with the accumulation of calcium in the ER [48]. HEK293T cells were treated with rapamycin and EGCG in the experiment. In the study, EGCG induced a delay in apoptotic cell death, also in the absence of GADD34 [48]. This is indicative of a shift in the balance of mTOR-AMPK pathways in the event of ER stress due to EGCG treatment to promote cell survival. The results indicate that EGCG activates autophagy via the mTOR-AMPK pathway, but ULK1 is essential in this process [48]. The research clearly indicates an important therapeutic role of EGCG, also in patients suffering from diseases associated with ER stress [48].

EGCG has anti-tumor activity through the regulation of autophagy via effects on reactive oxygen species (ROS) formation in many cancers. In lymphoma, EGCG inhibited the growth of BC-1 and BABL-1 cells, significantly inducing autophagy [49]. Studies in primary exudative lymphoma (PEL), induced by expansion of cells infected with human herpesvirus 8 (HHV8), have shown induction of apoptosis and cell cycle arrest as a result of the treatment with EGCG [49]. The results showed that EGCG increased Bax expression and *p53* activation, whereas previous studies showed chemical activation of *p53* to induce cell growth inhibition in the PEL model [49]. The available data also indicate an intense induction of autophagy as well as apoptosis in primary glioblastoma cell cultures [49]. The mechanisms were observed after the incubation with 500 μM EGCG. The authors claim that the use of even 100 nM EGCG activates endogenous mechanisms of protection of primary glioblastoma cell cultures and may have a chemopreventive effect [49].

Green tea catechins play a significant role in supporting the treatment [50] of breast [51], pancreatic [52], and lung cancers [53]. For instance, NSCLC A549 cell line was tested to determine the effect of green tea extract on autophagy [53]. The study used colorimetric assay for assessing cell metabolic activity MTT assay, cell death analysis by annexin V/PI assay, transmission electron microscopy (TEM), acridine orange staining (AO), and immunofluorescent labeling of LC3-I [53]. The authors suggested that autophagy was caused by the induction of cytoprotective autophagy by flavonoids present in green tea extracts [53]. Thus, dietary polyphenols alone may not have a significant effect on the growth of neoplastic cells, but will prove useful in combination therapy with cytostatic drugs to increase the effectiveness of oncological therapy [53]. Activating mutations in the EGFR gene in both wild-type and the mutant forms T790M, L858R, and ELREA are often found in NSCLC patients [54]. Experimental studies were carried out with the use of EGCG and EGFR-TKI (erlotinib) [54]. Positive results of the experiment such as in clarifying the differences in the bonds of both wild-type EGFR and mutant types, indicate that the use of polyphenols in the diet may affect the growth of cancer cells, which at the same time is an important aspect as adjunctive therapy [54].

As previously mentioned, EGFR plays a very important role in NSCLC [55]. The main green tea polyphenol, EGCG, was shown to overcome resistance to EGFR tyrosine kinase inhibitors such as gefitinib in wild-type A549 cells and 16HBE cells—the human bronchial epithelial cell line [55]. The results of the study showed the inhibition of ERK phosphorylation by combining gefitinib and EGCG through a reduction of p-ERK and p-MEK [55].

A significant amount of research is required to confirm the significant role of EGCG in autophagy [56]. One of the studies showed that the combination of a proteasome inhibitor bortezomib with EGCG enhances prostate cancer cell death mediated by an increase in ER stress [56]. EGCG caused an increase in autophagy through antagonizing the toxicity of bortezomib [56]. Subsequently, osteosarcoma stem cells were also investigated to develop the basis of anti-cancer drugs based on the results obtained with EGCG and doxyrubicin [57]. The results showed that the use of EGCG significantly reduced the expression of osteosarcoma stem cell markers, and, in particular, SOX2OT V7, by reducing Notch3/DLL3 signaling [57].

### 3.2. Oleuropein

**Figure 2:**
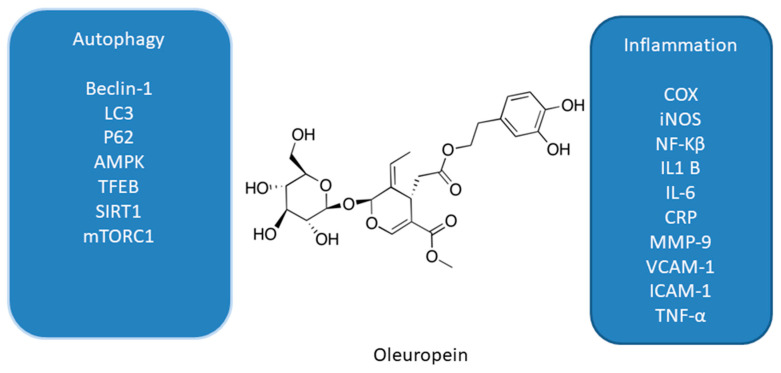
Effect of oleuropein on interplay between autophagy and inflammation [58,59,60,61,62,63,64,65].

It was also shown that leaves of European olive (*Olea europaea* L.), rich in phenolic compounds such as verbascoside, apigenin-7-glucoside, luteolin-7-glucoside, and the main polyphenol—oleuropein (OL, Figure 2), also have antioxidant, anti-inflammatory, and anti-tumor activities [58]. These features are associated with affecting proliferation and apoptosis by modulating expression of many signaling pathways. OL is a phenolic compound, chemically consisting of benzene-1,2-diol (hydroxytyrosol), 4-2-dyroxyethyl (polyphenols), and elenolic acid (secoiroid) [58].

The suggested anti-tumor mechanism of OL via the induction of autophagy has been recently confirmed in the estrogen receptor (ER)-positive MCF-7 and T47D breast cancer cell lines [59,60]. In addition to OL, hydroxytyrosol (HT) which is a metabolite of OL was also administered in the study [60]. MCF-7 and T47D cells were treated with OL and HT along with 3-methyladenine, which is an inhibitor of autophagy, with hepatocyte growth factor, or rapamycin, which is an agonist of autophagy [59]. The metastatic capacity and viability of the cells were assessed using the Western blot and transwell test. Depending on the dose, the viability of breast cancer cells decreased. Moreover, HT stronger than OL inhibited migration and invasion of triple-negative breast cancer cells by activating autophagy in the studied breast cancer cell lines [59]. Furthermore, it was showed that by suppressing autophagy, metastases of these cells can be inhibited [59].

OL was also used in a study determining the cellular and molecular activation mechanisms of autophagy [60,61,62,63]. One of the studies was conducted in an animal model and in cultured SH-SY5Y neuroblastoma cells. In the first study, the authors demonstrated a disruption of the autophagy cascade in SH-SY5Y cell line [60]. In a following work, cells were treated with 50 µM OL for 24 h and then assessed for *beclin-1* and AMPK phosphorylation. OL increased AMPK phosphorylation, an early marker of autophagy [61]. Moreover, OL use leads to mTOR inhibition through the induction of AMPK in the cortex of TgCRND8 mice [61].

The induction of AMPK-dependent autophagy by OL was noted in an experiment in an animal model of a C57BL/6J mouse [64]. Male and female mice were fed a high-fat and normal diet for eight weeks. Increased expression of *Beclin-1*, *LC3B*, and *p62/Sqstm1* genes was observed in the study. However, OL had no significant effect on the expression of the apoptotic proteins Bcl-2 and caspase 3 in this animal model [64].

The antiproliferative effect of OL and the induction of autophagy were confirmed in cultures of p-53 negative and androgen insensitive PC-3 human prostate cancer cells [65]. Moreover, the use of OL in the daily diet significantly reduced the dose of the cytotoxic doxorubicin—an anthracycline antibiotic with anti-cancer activity necessary to induce cell death [65], The results indicate a significant intensification of autophagy induction by OL, which was confirmed by the immunoblot analysis for LC3. However, it has to be stressed that the complex processes of autophagy induction due to OL use requires more research [65].

### 3.3. Pomegranate Extract and Punicalgin

**Figure 3:**
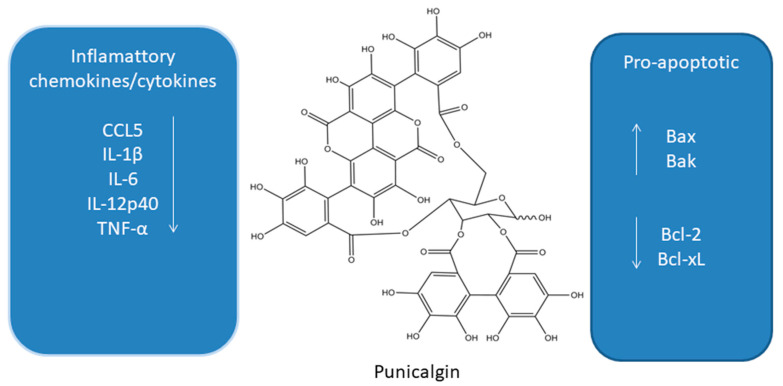
Influence of punicalgin on apoptosis and inflammatory cytokines [64,65,66,67,68,69,70,71,72,73,74,75,76].

Punicalgin (PUN, Figure 3) is a polyphenol obtained from pomegranate (*Punica granatum*). Depending on the form of antioxidants, it mainly contains anthocyanins, gallic acid, ellagic acid, ellagic tannins, and polyphenols. [34]. PUN is characterized by antioxidant activity and may inhibit lipoxygenases and cyclooxygenases. In addition, it has potential anti-cancer and chemopreventive effects. Both in vivo and in vitro studies confirm the effectiveness of PUN in the potential prevention of lung, breast, prostate, and colon cancers [64,65,66,67,68,69,70,71,72,73].

The chemopreventive effect of pomegranate polyphenols may be based on the action of a number of proteins and genes, which affect both the growth and progression of cancer [65]. Interestingly, compared with the activity of red wine containing resveratrol and green tea infusion, the antioxidant capacity of pomegranate juice was three times higher [62].

Glioblastoma is one of the most common brain tumors. Unfortunately, the prognosis for this type of cancer remains poor, due to a poor response to medical treatment [75]. Studies indicate that PUN at a concentration of 1–30 μg/ml induced cell death in U87MG glioma cell cultures [75]. It was demonstrated that PUN induced apoptosis by activating the caspase-9/caspase-3 cascade and by cleaving ADP-ribose in the human glioma cell line. In addition, PUN increased autophagosome formation. Since PUN increased the phosphorylation of *p27* T198 and of AMP, it was suggested that cell death mediated by PUN occurs via the apoptotic and autophagy pathways [75].

Pomegranate extract (PE) polyphenols show autophagy modulating effects in, inter alia, human syncytiotrophoblast and neuronal human neuroblastoma SY5Y cells [76]. PE activates the upregulation of both lysosomal and autophagosomal compartments, as well as causing autophagosome replacement and induction of autophagosome formation [76]. Importantly, TFEB activation is induced by the use of PE in a cytosolic Ca^2+^-dependent manner, but has no effect on ERK1/2, AKT and mTOR calcineurin signaling. Moreover, PE stimulated PINK1-Parksi mitophagy during of mitochondrial stress [76].

### 3.4. Apigenin—An Antioxidant from Acerola Fruit

Acerola (*Malpighia glabra*) is also known as the Barbados Cherry [40]. Its edible part are small red fruits with juicy flesh. Acerola is a rich source of vitamin C, beneficial to our health [40,69]. Acerola, apart from vitamin C and bioflavonoids, also contains pantothenic acid (vitamin B5), magnesium, potassium, thiamine, niacin, riboflavin, and vitamin A [40,77].

**Figure 4:**
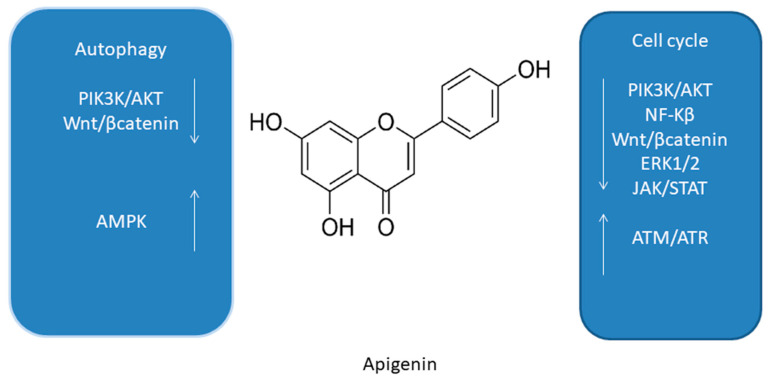
Summary of molecular cell cycle and autophagy signaling pathways of apigenin [66,71,78,79,80].

One of the major flavonoids contained in acerola is apigenin (AP, Figure 4) [66]. A number of studies, both in vitro and in vivo, confirmed the chemopreventive effect of AP [78], which occurred due to the inhibition of inflammation and angiogenesis, a significant delay in cell proliferation, a cellular response to oxidative stress, and the induction of apoptosis and autophagy [78].

In one of the studies the influence of AP on the modulation of AMPK in human keratinocytes (HaCaT cell line and primary normal human epidermal keratinocytes) was investigated [79]. AP inhibited the mTOR signaling pathway by inducing autophagy via the activation of AMPK [71]. In addition, results of another study suggest that AP causes autophagy in C6 glioma cells [68]. Treatment of C6 cells resulted in reduced AMPK phosphorylation and, importantly, autophagy was AMPK/mTOR independent [80]. Importantly, it is worth emphasizing that the difference in experimental studies using C6 cells and glioma cells differs in particular in the resistance of HaCaT cells to apoptosis, which can be explained by mutations within the pro-apoptotic *p53* protein alleles [68,80].

### 3.5. Resveratrol

**Figure 5:**
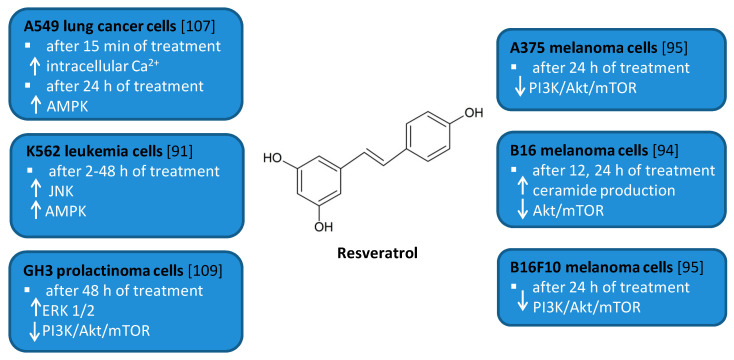
Cell signaling pathways triggered by resveratrol in selected cancer cell lines. ↑ activation/increase; ↓ inhibition/decrease.

Resveratrol (RSV) (trans-3,4′,5-trihydroxystilbene, Figure 5) is a naturally occurring polyphenol found in berries, peanuts, grapes, and wine [81,82,83]. It has attracted much attention due to the beneficial biological effects related to its anti-oxidant, anti-cancer, anti-inflammatory, anti-diabetic, and cardioprotective activities [84,85,86,87,88]. RSV has been shown to modulate autophagy in many cancer cell lines including leukemia, melanoma, glioma as well as renal, esophageal, liver, colon, prostate, breast, ovarian, oral, and lung cancer cell lines [89,90,91,92,93,94,95,96,97,98,99,100,101,102,103,104,105,106,107,108].

Several in vitro studies indicated that RSV is able to induce autophagy that plays a pro-survival role and may serve as a resistance mechanism against apoptotic cell death [94,109]. For example, RSV triggered protective autophagy rough the ceramide accumulation and inhibition of Akt/mTOR pathway in B16 melanoma cells [94]. Inhibition of autophagy in B16 cells markedly increased RSV-induced apoptosis suggesting that RSV-induced autophagy plays a pro-survival role. In addition, the protective role of autophagy was demonstrated in resveratrol-treated U251 human glioma cells [96]. Similar results concerning the pro-survival role of RSV-induced autophagy were observed in GH3 cell line derived from rat pituitary tumor cells [109]. RSV led to the upregulation of ERK1/2 and the downregulation of PI3K/Akt and mTOR phosphorylation in prolactinoma GH3 cells. In some experimental models, RSV-induced autophagy contributed to the elimination of cancer cells [91,95,107]. RSV triggered autophagic cell death in imatinib-sensitive and imatinib-resistant K562 chronic myelogenous leukemia cells [91]. It led to the JNK-mediated p62/SQSTM1 expression and it activated AMPK. Thus, both JNK and AMPK pathways were involved in autophagic elimination in K562 cells. The activation of AMPK and JNK participated independently with the initiation and elongation steps of autophagy, respectively. RSV also suppressed the growth of A375 human melanoma cells and B16 F10 murine melanoma cells by promoting autophagy and inhibiting the PI3K/AKT/mTOR signaling pathway [95]. Moreover, it has also been found to induce autophagic cell death in A549 human lung adenocarcinoma cells via Ca^2+^/AMPK-mTOR signaling pathway [107].

#### 3.6. Pterostilbene

**Figure 6:**
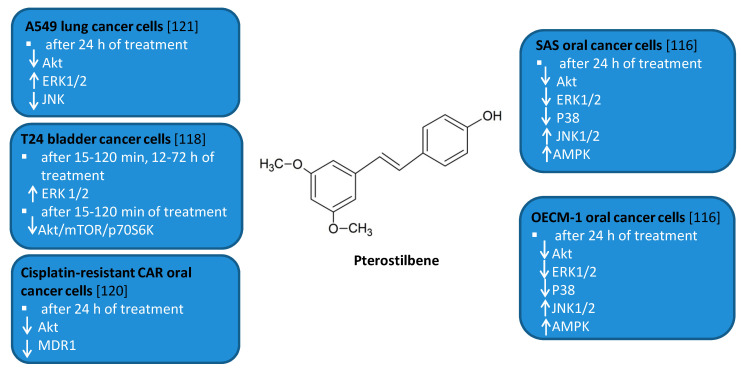
Cell signaling pathways triggered by pterostilbene in selected cancer cell lines. ↑ activation/upregulation; ↓ inhibition/downregulation.

Pterostilbene (PTS, trans-3,5-dimethoxy-4’-hydroxystilbene, Figure 6) is a natural polyphenolic compound found in fruits such as blueberries and grapes [81,110]. It is a structural analog of RSV. It has been demonstrated to have anti-oxidant, anti-inflammatory, anti-cancer, and anti-diabetic properties [81,110,111,112,113,114].

PTS has been shown to modulate autophagy in leukemia, oral, liver, bladder, breast, and lung cancer cell lines [115,116,117,118,119,120,121]. Both autophagic and apoptotic markers were detected after treatment of HL60 human leukemia cells with PTS. [115]. PTS induced accumulation of large autophagic vacuoles in HL60 cells followed by cell death. Autophagy inhibitor 3MA did not decrease cell death of HL60 cells. Long term exposure of MCF-7 human breast cancer cells to PTS resulted in the activation of autophagy and differentiation of MCF-7 cells into cells with normal epithelial cell-like morphology [119]. PTS-induced autophagy in MCF-7 cells was blocked by PI3K inhibitor 3MA, PI3Kinhibitor wortmannin, and MEK inhibitor PD98059. Moreover, 3MA protected MCF-7 cells from cytotoxic effects of this compound. PTS was found to induce autophagy in human tongue squamous carcinoma (SAS) and human oral cavity squamous cell carcinoma (OECM-1) human oral cancer cells by inhibition of Akt, ERK1/2, p38, and activation of the JNK1/2 pathway as well as AMPK activation [116]. 3MA increased the cell viability of PTS-treated SAS and OECM-1 cells. However, it also led to the slight increase in the levels of cleaved caspase-3, -8, and -9. Other authors reported that PTS induced ER stress and autophagy-dependent cell death of Huh-7 and SK-Hep-1 human liver cancer cells [117]. Treatment of Huh-7 and SK-Hep-1 cells with PTS in combination with an eIF2α phosphatase inhibitor Sal enhanced autophagy-dependent cell death through the involvement of peIF2α/ATF4/LC3 pathway. 3MA partially reduced PTS cytotoxicity in these cells but the expression of cleaved caspase -3 did not decrease. Moreover, this polyphenol has also been reported to induce autophagic and apoptotic cell death in cisplatin-resistant human oral cancer (CAR) cells by the mechanism involving AKT-mediated multidrug resistance protein 1 (MDR1) suppression [120]. PTS induced autophagy in sensitive and chemoresistant T24 human bladder cancer cells by inhibition of AKT/mTOR/p70S6K pathway and activation of ERK1/2 pathway [118]. Inhibition of autophagy by 3MA, Baf A1 as well as BECN1 shRNA or ERK1/2 shRNA transfection enhanced apoptosis in PTS-treated T24 cells. Another study showed that PTS induced autophagy in chemosensitive and chemoresistant A549 human lung cancer cells by inhibition of the Akt and JNK pathways and activation of ERK1/2 [121]. Autophagy inhibitors 3MA and bafilomycin A1 as well as *beclin-1* siRNA enhanced apoptosis in PTS-treated A549 cells.

### 3.7. Curcumin

**Figure 7:**
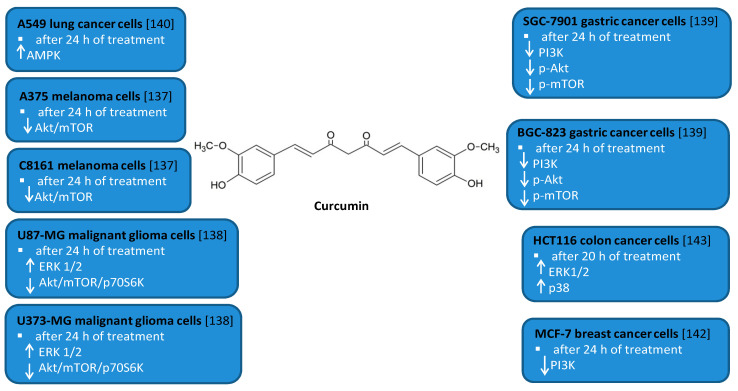
Cell signaling pathways triggered by curcumin in selected cancer cell lines. ↑ activation; ↓ inhibition.

Curcumin (diferuloylmethane, 1,7-bis(4-hydroxy-3-methoxyphenyl)hepta-1,6-diene-3,5-dione, CR, Figure 7) is a natural polyphenol derived from rhizomes of *Curcuma longa*—the perennial herb commonly known as turmeric [122]. Turmeric is used in human diets as a culinary spice [123]. Accumulating evidence shows that CR possesses anti-oxidant [124,125,126,127], anti-inflammatory, and anti-cancer properties [124,125,126,127]. It has been shown to be highly cytotoxic against several cancer cell lines such as lung, gastric, pancreatic, colon, breast, prostate cancer cell lines, or leukemia and lymphoma cell lines [128,129,130,131,132,133,134,135].

Numerous studies have indicated that CR is able to modulate autophagy in cancer cell lines including leukemia, melanoma, glioma as well as gastric, colon, prostate, and lung cancer cell lines [124,125,136,137,138,139,140,141,142,143]. CR induced autophagy in SGC-7901 and BGC-823 human gastric cancer cells [139]. It led to the downregulation of the expression levels of PI3K, p-Akt, and p-mTOR proteins. Treatment with autophagy inhibitor hydroxychloroquine promoted CR-induced apoptosis in both gastric cancer cell lines. CR also induced apoptosis and protective autophagy in DU145 and PC3 human prostate cancer cells, which are at least partially dependent on its iron-chelating properties [141]. Other studies showed that CR triggered autophagy and apoptosis in MCF-7 human breast cancer cells and PI3K inhibitor LY294002 enhanced CR-induced apoptosis [142]. CR induced autophagy in A549 human lung cancer cells via activating the AMPK signaling pathway [140]. Moreover, this polyphenolic compound induced autophagic cell death in U87-MG and U373-MG malignant glioma cells [138]. CR inhibited Akt/mTOR/p70S6K pathway and activated ERK1/2 pathway in these cells. In addition, in the subcutaneous xenograft model of U87-MG cells it inhibited tumor growth and induced autophagy [138]. Another study demonstrated that CR induced autophagy and inhibited proliferation and invasion of A375 and C8161 human melanoma cells by downregulating AKT/mTOR signaling pathway [137]. Other authors reported CR-induced production of reactive oxygen species in HCT116 human colon cancer cells followed by autophagic cell death [143]. CR induced ROS-dependent activation of ERK1/2 and p38 MAPK signaling pathway but this pathway is not critical to induce autophagy in HCT116 cells. Moreover, treatment of K562 human myeloid leukemia cells with CR also resulted in apoptotic and autophagic cell death [136].

Accumulating evidence indicates that administration of CR at high doses may be associated with side effects [144]. Human studies have demonstrated that CR administered at doses ranging from 0.45 to 3.6 g daily for up to 4 months led to some adverse effects such as nausea and diarrhea and caused an increase in serum alkaline phosphatase and serum lactate dehydrogenase [145]. CR has also been shown to be toxic to normal human dermal fibroblasts [146]. Treatment of these cells with 10 µM CR resulted in the G2/M-phase cell cycle arrest and apoptosis.

## 4. Conclusions

Accumulating evidence suggests that autophagy plays a dual role in cancer [6]. On the one hand, autophagy may play a cytoprotective role [7], and on the other hand, it may promote the development of tumors [8,9]. It is worth emphasizing that autophagy also may play a role in promoting chemoresistance [30,31,32]. The impact of autophagy on the reduction or progression of neoplastic processes is strictly dependent on the type and stage of the tumor, microenvironment, applied therapeutics, or the genetic context [8,9].

Moreover, autophagy plays an important role in cell death—it is known that the mechanism of autophagy can induce autophagy-induced cell death, but it is also possible to induce cell death in a manner independent of necrosis or apoptosis [25].

Polyphenols play an important role in modulating autophagy [38]. A number of in vitro and in vivo studies confirmed the anti-inflammatory and antioxidant properties of polyphenolic compounds as well as their ability to induce autophagic death in cancer cells [35]. Moreover, they have been suggested to play an important role in the prevention of cancer diseases [66,71,78]. The biologically active ingredients of fruits and vegetables, described in this review, such as the main catechin of green tea, EGCG [42,43,44,45,46,47,48,49,50], oleuropein [49,50,51,52,53], punicalgin [39,49,50,51,52,53,54,55,56,57,58,59,60,61,62,63,64,65,66,67,68,69,70,71,72,73,74,75], apigenin [40,77,78,79,80], resveratrol [81,82,83,84,85,86,87,88,89,90,91,92,93,94,95,96,97,98,99,100,101,102,103,104,105,106,107,108,109], pterostilbene [81,110,111,112,113,114,115,116,117,118,119,120,121], and curcumin [122,123,124,125,126,127,128,129,130,131,132,133,134,135,136,137,138,139,140,141,142,143,144,145], seem to be valuable and promising agents in cancer chemoprevention.

## Figures and Tables

**Table 1:** The examples of dietary compounds rich in antioxidants.

Dietary Source	Classification of Polyphenols
Pomegranate [39]	Tannins
	Anthocyanins
	Ellagic acid
	Elagotannins such as punicalgin and munialin
Acerola [40]	Chlorogenic acid
	P-coumaric acid
	Ferulic acid
	Kaempferol
	Luteolin
	Routine
	Apigenin
	Anthocyanins (cyanidin, delphinidin 3β-D-glucoside, phloretin, peonidin)
Grapes [41]	Resveratrol
	Quercetin
	Ellagic acid
	Kaempferol
Green tea catechins [42]	(−)-epigallocatechin-3-gallate (EGCG)
	(−)-epicatechin-3-gallate (ECG)
	(−)-epigallocatechin (EGC)
	(−)-epicatechin (EC)
Wine [43]	Resveratrol
	5.2. Piceatannol
	Quercetin
	Kaempferol
	Myricetin
	(+)-catechin
	(−)-EC
	(−)-ECG
	(−)-EGC
